# CRT in Patients with Heart Failure: Time Course of Perfusion and Wall Motion Changes

**DOI:** 10.4061/2010/981064

**Published:** 2010-06-24

**Authors:** Alessia Gimelli, Paolo Frumento, Guido Valle, Mario Stanislao, Umberto Startari, Marcello Piacenti, Paolo Marzullo

**Affiliations:** ^1^Gabriele Monasterio Foundation, CNR, 56124 Pisa, Italy; ^2^CNR Institute of Clinical Physiology, Via Moruzzi, 1, 56124 Pisa, Italy; ^3^Department of Statistics, Univeristy of Florence, 50121 Florence, Italy; ^4^Scientific Institute “Casa Sollievo della Sofferenza”, 71013 San Giovanni Rotondo, Italy

## Abstract

In patients treated with CRT no data relative to the relationship between regional wall motion and perfusion and reverse remodelling of the left ventricle at short and medium term followup were available. To this aim, 36 heart failure patients were studied by G-SPECT before (T0), within 2 months (T1) and 6 months (T2) after CRT. A clinical followup was completed for 36 months. In 30/36 patients there was an improvement of NYHA Class at T1 that persisted at T2. G-SPECT showed significant improvement of perfusion at T1 in 92% of patients without further changes at T2. A reduction of LV volumes, an increase of EF and an improvement of regional wall motion and thickening were observed at T1 versus baseline, with only minor changes at T2. Moreover, baseline extension of perfusion defects was scarcely correlated with improvement after CRT. Finally, end diastolic volume, perfusion defect and diabetes mellitus were independent predictors of survival. The main effects of CRT on regional myocardial perfusion and wall motion are obtained within 2 months. Volume overload modulates recovery of ventricular function independently of reperfusion and, with extension of perfusion abnormalities and diabetes were independent predictors of survival during followup.

## 1. Introduction

Progression of left ventricular dysfunction to heart failure with low-ejection fraction (EF) is frequently accompanied by impaired electromechanical coupling, which may further diminish effective ventricular systolic function. Cardiac resynchronisation therapy (CRT) has been proposed as treatment of patients with idiopathic as well as ischemic left ventricular dysfunction with drug-refractory heart failure and intra- and interventricular conduction delay [1, 2]. The physiological and mechanical effects of CRT are well described in literature in [3, 4] as well as the changes of regional myocardial perfusion and metabolism that contribute to the benefits observed in [5, 6]. However, despite a significant number of published studies [2–9], few data are available regarding time of recovery of ventricular function. In fact, studied populations were not comparable in terms of followup time as well as in terms of imaging variables analysed for outcome. 

For this purpose, we studied a group of 36 patients submitted to CRT in order to identify the time course of perfusion and wall motion changes at short- and medium-term followup. Rest myocardial gated single photon emission computed tomography (G-SPECT) was used to detect simultaneously perfusion, regional wall motion, EF and volumes. All patients were followed up for 36 months.

## 2. Study Design

Thirty-six consecutive patients, 14 with ischemic (CAD) and 22 patients with idiopathic (DCM) cardiomyopathy, scheduled for CRT due to congestive heart failure, class NYHA III and IV, left bundle branch block with QRS ≥ to 120 ms, left ventricular ejection fraction (EF) ≤35%, and persisting symptoms despite maximal medical therapy, were prospectively evaluated. Exclusion criteria were permanent atrial fibrillation, significant valvular heart disease, restrictive or hypertrophic cardiomyopathy, suspected acute myocarditis, acute coronary syndrome (<3 months), and severe chronic obstructive pulmonary disease. Table 1 summarizes clinical and demographic characteristics of the patients before entering the study. After enrolment, gated SPECT were obtained at rest before implantation (T0), repeated within 60 days (T1) and after 6 months (T2). At enrolment all patients were on optimal medical regimen that was unchanged at the time of followup studies; those patients requiring significant changes in diuretics, ACE inhibitors, digitalis and beta-blockers after CRT were excluded from the study. In 16/36 patients, the CRT treatment was an upgrading of previous pacemaker (timing of previous implantation: 22 ± 7 months). At baseline, 20/36 patients had a normal sinus rhythm while those 16 who were upgraded had a pacemaker-induced rhythm (7 with purely ventricular rhythm and 9 with residual atrial activity). The study was approved by the local ethical committee. All patients signed an informed consent to participate to the study.

### 2.1. Device Implantation

A permanent biventricular transvenous pacing system was implanted in the patients (Guidant—Renewal 2 in 19 patients and Medtronic-Insync III in the remaining 17). Devices were programmed to maximise biventricular pacing throughout the range of expected patients activity and to minimise power output to prolong battery life. Further optimisation of atrio-ventricular delay was performed using Doppler transmitral flow to provide the maximum left ventricular filling time without compromising cardiac resynchronisation. The AV delay was set at a value that provided maximum separation of the E and A waves, in order to select the shortest AV delay without compromising the left atrial contribution to the left ventricular filling.

### 2.2. Gated SPECT

Gated SPECT acquisition began 45 to 60 min after 99m Tc Tetrofosmin injection at rest (up to 450 MBq). A double-head gamma camera equipped with high-resolution collimators was used. A 64 × 64 matrix, 32 projections, 40 seconds projections, 16 frames/cycle protocol were applied in association with appropriate energy photopeaks. All studies were reconstructed using filtered backprojection without attenuation or scatter correction. An operator independent analysis of regional wall motion and wall thickening was performed using previously validated software for quantitative gated SPECT analysis (QGS, Cedars Sinai, US), providing scores of regional wall motion (from 0 = normal to 5 = dyskinesis) and wall thickening (from 0 = normal to 3 = absent) in a 20-segment polar map. Summed motion scores (SMSs) and summed thickening scores (STSs) were automatically calculated by adding regional scores. Left ventricular end diastolic volume (EDVi) and end systolic volume (ESVi) were calculated using the same programme and normalized to the body surface area. Regional perfusion was assessed qualitatively by the consensus of two skilled observers, comparing baseline and followup images and identifying those segments with improvement or reduction of perfusion using a 5-point score (from 0 = normal to 4 absent perfusion) in the same 20-segment polar map [10]. Moreover, regional perfusion was assessed also by means of a quantitative perfusion score (QPS, Cedars Sinai, US), providing % regional uptake in the same 20-segment polar map. By adding the 20-segment scores, a summed rest scores (SRSs) were automatically calculated. A SRS < 4 was considered as normal, 4 to 8 as mild to moderate and >8 as extensive perfusion defects [11]. 

### 2.3. Followup

According to the followup program of our Institute, one year after CRT, each patient was evaluated by medical interview, physical examination and 12-lead ECG. Furthermore, patients were followedup for additional 12 to 36 months by telephone calls or mail questionnaires. We defined as cardiac events: cardiac death, nonfatal myocardial infarction, coronary revascularization by percutaneous coronary interventions or bypass surgery, and hospitalisation for cardiac ischemic events. No one of the enrolled patients was lost to followup. In particular, 4 patients who could be initially considered for the enrollment were excluded from the studied population because two patients died up to 60 days of followup and two up to 6 months. All patients were informed of the observational and investigative nature of the study and gave their written informed consent.

## 3. Statistical Analysis

Results are expressed as mean ± SD. Variables were compared with the Students *t*-test for paired or unpaired samples, and with the ANOVA test. A *P* value < .05 was considered statistically significant. Linear regression analysis was performed to evaluate relations between variables. Chi-square test was used to test the independence between categorical variables.

All quantitative G-SPECT variables (EF, EDVi, ESVi, SMS, STS, and SRS) were examined to test the differences and correlations between T0, T1, and T2. 

The independent predictors of survival free from cardiac death were identified using multivariate Cox regression analysis, and including all the variables collected. A backward elimination procedure was used to select the independent variables: that is, those resulted to be significant.

## 4. Results

### 4.1. Clinical and Electrocardiographic Findings

Implantation was successful in all patients and no complications were reported. Left ventricular pacing leads were positioned in lateral (18 patients), anterior (6 patients), or posterior (12 patients) cardiac veins. All but three patients showed a NHYA class improvement at T1 that was confirmed at T2.

### 4.2. Differences between CAD and DCM Patients

When clinical and quantitative variables were examined in CAD and DCM patients, respectively, no differences was found between baseline NYHA Class (*P* = .215), EDVi (*P* = .977), ESVi (*P* = .910), EF (*P* = .210), SMS (*P* = .734), and STS (*P* = .812). As expected, the only statistically different variable was SRS (25.5 versus 7.8, *P* = .0001, resp. in CAD and DCM patients).

### 4.3. Time to Recovery and Correlation between Variables

The analysis of all G-SPECT variables showed significant differences between T0 and T1 as well as T2 (Table 2). However, the major effects were found in the first 2 months, while at 6 months there were only minor changes (Tables 3(a) and 3(b)). 

The volume reduction observed at T1 was inversely correlated to the volume overload at T0 (*P* = .0025). Those patients with the highest volume overload showed less ventricular reverse remodelling and improvement of clinical variables. In particular, 11 patients (average EDVi: 201 ± 25 ml/m^2^ and ESVi 172 ± 18 ml/m^2^ at T0) showed no reverse remodelling or improvement in clinical variables (average EDVi: 205 ± 19 ml/m^2^ and ESVi 176 ± 13 ml/m^2^ at T1, *P* = ns versus T0). On the contrary, at T2 perfusion and wall motion data reflected the results obtained in the first two months, without significant changes (*P* = .455 and *P* = .348, resp., for perfusion and wall motion abnormalities at T2 versus T1). The same results were obtained when CAD and DCM patients were analysed separately.

### 4.4. Perfusion Analysis

T1 qualitative perfusion analysis indicated that in all but 3 patients there was a significant improvement of tracer uptake at rest (mean value of qualitative score in the polar map 14 versus 10.5 and 11 at T0 versus T1 and T2, *P* = .0035, resp.). The segments that benefit most from CRT in terms of perfusion were anterior septum (24 patients), inferior septum (22 patients), inferior wall (generally in the distal portion, 16 patients), anterior wall (7 patients), while in only 2 patients there was an improvement in posterior and lateral wall. All but 3 patients showed relationship between site of improvement of tracer uptake and pacing lead position. However in all these patients improvement of perfusion was detected also in a control area. No significant differences were observed between T1 and T2 at qualitative analysis. The same results were obtained with quantitative analysis (Table 2). 

The qualitative as well as quantitative analysis of SRS indicated that perfusion parameters were not correlated with other variables in predicting patient's outcome. As a matter of fact, perfusion score, obtained from all 36 patients, at T0 was scarcely correlated with the volume reduction and improvement of function at T1 and at T2. 

No significant differences were observed when the same variables were analysed separately in CAD and DCM patients.

### 4.5. Followup

Three patients (2 male and 1 female) died for end-stage heart failure during followup (two of them up to 12 months and one after 24 months). 

Using Cox proportional hazard regression analysis, a EDVi (Hazard ratio 1.02; 95% confidence Intervals 1.00–1.24; *χ* ^2^ = 31.12; *P* = .00002), SRS (Hazard ratio 1.15; 95% confidence Intervals 1.05–1.29; *χ* ^2^ = 27.30; *P* = .00003), and diabetes mellitus (Hazard ratio 2.51; 95% confidence Intervals 1.18–4.75; *χ* ^2^ = 18.76; *P* = .0013) were independent predictors of survival during followup. No correlation was found between the presence of coronary artery disease and cardiac events during followup (*P* = .19).

## 5. Discussion

Previous studies demonstrated that CRT may improve cardiac function and clinical status in patients with moderate to severe heart failure and a prolonged QRS interval [3–5]. However, despite the presence of several published studies, few data are available regarding the time sequence of improvement after resynchronization and the behaviour of functional and perfusion variables in these patients before and after CRT. 

Our results indicate that the main effects of CRT on global function and regional myocardial perfusion are obtained within 2 months and are stable over a period of 6 months. Moreover, volume overload modulates recovery of regional and global ventricular function independently of reperfusion both at short- and medium-term followup. Finally, reperfusion is a common finding after CRT and it is not coupled to recovery of function at short- as well as medium-term followup. On this basis, gated SPECT provides diagnostic and pathophysiological information that should be carefully evaluated in heart failure patients suitable for CRT.

### 5.1. Time to Recovery

One of the main results obtained by this study is relative to time to recovery after CRT. In our knowledge, this is the first study showing that CRT benefits are evident within 60 days after resynchronisation and thereafter remain stable or follow the trend of the first two months over a range of 6 months. This result could be helpful in the strategy of the followup examinations of resynchronized patients. A possible explanation of this phenomenon could be due to an acute mechanical effect of CRT [3, 4] with a subsequent improving of the hemodynamic, that is particularly evident in patients with poor or moderate volume overload. On the contrary, benefit of CRT is limited by the degree of volume overload like in other categories of patients such as those with viable myocardium undergoing coronary revascularization or with valvular diseases undergoing surgery [12, 13]. In patients without improvement after CRT, recovery of function and perfusion is not observed at the first followup control as well as after 6 months.

### 5.2. CRT Efficacy

The multiple effects of CRT include complex perfusion and metabolic changes that may explain some of the short- and medium-term benefits independently of wall motion or hemodynamic changes. Our study confirms previous clinical observations showing an improvement in heart failure symptoms, also in patients without significant volume reduction, and introduces the concept of reperfusion not limited to the septal but extended to the anterior, inferior and lateral wall. As a matter of fact, reperfusion is independent not only by symptoms but also by recovery of function, indicating that CRT works on a wide spectrum that includes perfusion as well as function. Probably, the “mismatch” of reperfusion without any apparent improvement in wall motion could identify those patients in whom the clinical improvement is not obtained merely by a volumetric resynchronisation but mostly by a normalization of myocardial blood flow.

### 5.3. Followup

Despite the small number of patients, our data demonstrated that volume overload and extension of perfusion defects were the most important variables in determining event-free survival in patients with as well as in patients without coronary artery disease. The absence of a significant difference in cardiac mortality between the two groups at 3 years, was a very interesting result. Thus, in clinical practice, the need for additional imaging variables, such as extension of scar and volumes, seems to be necessary before CRT, according to standard risk stratification.

### 5.4. Limitations

The study population includes 36 patients and thus some of our conclusions may suffer from this limited group. The difficulty of maintaining the same medical treatment from baseline to followup and the occurrence of death in two patients with severe volume overload reduced the study group but at the same time avoided any interference from medical therapy, a variable frequently underestimated in followup studies. Perfusion was evaluated only at rest, and thus all considerations concerning Tetrofosmin uptake linearity with coronary blood flow should not limit our results. The limits of QGS underestimation of absolute volumes in severe dysfunctioning patients should not affect our conclusions since all patients were processed in the same way before and after CRT. The limit of correctly identifying myocardial borders in such dilated hearts was always checked and corrected with operator manual experience. 

Finally, a control group of dysfunctioning patients who did not undergo CRT but who were submitted to gated SPECT has not been included in the study because of ethical implication in order to reduce radiation exposure.

## 6. Conclusions

This study demonstrated that the main effects of CRT on regional myocardial perfusion and wall motion are obtained within 2 months and are stable over a period of 6 months. Volume overload modulates recovery of regional and global ventricular function independently of reperfusion both at short- and medium-term followup. Moreover, extension of perfusion defects as well as volume overload were the most important variable in determining event free survival in the followup. Finally, this study also demonstrates that CRT works on a wide spectrum that ranges from QRS to perfusion, and that some unexplained beneficial effects may be due to reperfusion beyond mechanical effects.

## Figures and Tables

**Table 1:** Clinical and Demographic Characteristics of patients. EF: ejection fraction, MI: myocardial infarction, NYHA: New York Heart Association, EDVi: end diastolic volume index, ESVi: end systolic volume index, and Q: flow rate.

	*n* = 36
Mean age (years)	70 ± 8
Sex (male)	24 (67%)
EF	22.2 ± 3%
Diabetes	21 (58%)
Hypertension	25 (79%)
MI site	Anterior: 8 (60%)
	Inferior: 6 (40%)
NYHA class	3.1 ± 0.4
EDVi	141.2 ± 60.5 ml/m^2^
ESVi	129.2 ± 50.6 ml/m^2^
Motion extension	63 ± 19%
Summed Motion Score	48.9 ± 11.5
Summed Rest Score	14.5 ± 12.6
Summed Thickening score	34.4 ± 6.1
Q Aortic	158 ± 46
Q Pulmonary	114 ± 36

**Table 2:** Value of scintigraphic variable obtained at baseline and at short- and medium-term of followup. T0: time 0, T1: first step of followup, T2: second step of followup. EDVi: end diastolic volume index, ESVi: end systolic volume index, SMS: summed motion score, STS: summed thickening score, SRS: summed rest score, and EF: ejection fraction.

Variables (mean)	T0	T1	T2	Difference T1-T2
EDVi	167.75	144.66***	138.79***	−5.87 n.s.
ESVi	133.97	112.55***	105.41***	−7.14 n.s.
SMS	48.91	40.42***	38.47***	−1.95 n.s.
STS	34.41	31.25**	31.11**	−0.14 n.s.
SRS	14.69	11.58**	12.06**	+0.48 n.s.
EF	22.27	27.38***	28.64***	+1.26 n.s.

**P < .05 versus T0; ***P < .01 versus T0; n.s.: not significant.

**Table:** (a) Percent of variable variations at T1.

% of variations T0 versus T1						
Corr.	EDVi	ESVi	SMS	STS	SRS	FE
EDVi	1					
ESVi	0.945***	1				
SMS	0.407**	0.578***	1			
STS	0.556***	0.716***	0.864***	1		
SRS	0.417**	0.391**	—	—	1	
FE	−0.699***	−0.819***	−0.740***	−0.799***	—	1

**Table:** (b) Percent of variable variations between T1 and T2.

% of variations T1 versus T2						
Corr.	EDVi	ESVi	SMS	STS	SRS	FE
EDVi	1					
ESVi	0.801***	1				
SMS	0.330*	0.567***	1			
STS	0.429***	0.485***	0.631***	1		
SRS	—	—	0.377**	0.500***	1	
FE	−0.473***	−0.676***	−0.792***	−0.403**	—	1

***P < ,01 **P < ,05 *P < ,1.
